# Development and validation of the Chinese version of the self-management support scale for kidney transplant recipients

**DOI:** 10.1186/s12912-023-01269-x

**Published:** 2023-04-18

**Authors:** Keke Lin, Changyun Wei, Jiaqi Li, Xuejie Guo, Fengli Gao, Peiyu Zhao, Hongxia Liu

**Affiliations:** 1 School of Nursing, Beijing University of Chinese Medicine, Beijing, China; 2 Nursing Department, Beijing Chao-Yang Hospital, Capital Medical University, Beijing, China; 3 Nursing Department, China-Japan Friendship Hospital, Beijing, China

**Keywords:** Self-management support, Kidney transplant, Instrument development, Validity, Reliability

## Abstract

**Background:**

Providing self-management support to kidney transplant recipients is essential. However, a scale to identify the self-management support they have received is lacking. The purpose of this study is to develop a Self-management Support Scale for Kidney Transplant Recipients (SMSSKTR) and test its psychometric properties.

**Methods:**

This is an instrument development and validation study, which has a three-stage cross-sectional design. In Stage 1, a preliminary item pool was formed using a literature review, semi-structured interviews, and the Delphi method. In Stage 2, six experts were invited to assess content validity. A convenience sample of 313 participants was used to explore the factor structure by using exploratory factor analysis. The test-retest reliability was assessed using the intra-class correlation coefficient (ICC). In Stage 3, two hundred and sixty-five participants were recruited to validate the factor structure by using confirmatory factor analysis. Convergent validity was examined using Spearman’s correlation coefficient. Cronbach’s alpha coefficient and corrected item-total correlation coefficient were used to test the reliability of the entire scale and its dimensions. The study was reported according to the STARD and GRRAS checklists.

**Results:**

An initial 40-item scale was developed in Stage 1. In Stage 2, three factors with 22 items emerged from the exploratory factor analysis: instrumental support, psychosocial support, and relational support. The content validity index of the scale was 0.97. The intra-class correlation coefficient for the entire scale and the subscales were 0.915, 0.771, 0.896, and 0.832, respectively. In Stage 3, the confirmatory factor analysis indicated that the three-factor model had a good fit. The score of the scale was positively associated with that of the Self-Management Scale of Renal Transplant Recipients (*r* = 0.532). Cronbach’s alpha was 0.959 for the entire scale and 0.956–0.958 for the three subscales. The corrected item-total correlation coefficient ranged from 0.62 to 0.82.

**Conclusion:**

The 22-item SMSSKTR has sufficient psychometric properties to assess the self-management support they have received, which has not been measured before.

**Supplementary Information:**

The online version contains supplementary material available at 10.1186/s12912-023-01269-x.

## Background

Kidney transplantation has superior effects on decreasing patients’ mortality and improving quality of life for patients with end-stage kidney disease [1]. However, kidney transplant recipients (KTRs) are at high risk of rejection and complications [2]. They need to adhere to complex medication treatment regimens [3], monitor their physical conditions, and perform regular and lifelong follow-up visits to specialists [4]. Moreover, they need to adapt to changes in social roles and relationships, manage emotions, and establish new perspectives in life [5, 6]. Therefore, KTRs face various challenges in completing the self-management tasks after kidney transplantation. KTRs reported difficulties in self-management and lacked guidance on effective self-management knowledge and skills [7, 8]. Ineffective self-management compromises the quality of life, increases medical costs, and affects the survival rate of KTRs [9–11]. Therefore, self-management support is necessary for KTRs to better perform self-management tasks and improve their health outcomes.

The concept of self-management support was first proposed by Creer [12] and was widely used in the management of chronic diseases, such as diabetes, cardiovascular and cerebrovascular diseases, organ transplantation, hypertension, and cancer. However, self-management support for KTRs has not been clearly defined. According to the definition of diabetes self-management support by the American Diabetes Association (ADA) [13], we could define the self-management support of KTRs as activities that help KTRs achieve and maintain their self-management behaviors. The types of support mainly include instrumental support, that is, disease-related medical management; psychosocial support, which refers to emotional and psychological resources needed to manage the disease and relational support, the beneficial interaction with others [14]. Self-management support comes from a wide range of sources, including medical staff, disease management educators, community health service personnel, governments, organizations, families, relatives, friends and other KTRs [14]. It mainly includes the development of behavioural objectives, education about self-management knowledge, good medication management, psychosocial support, economic and medical policy support, and regular follow-up and examination reminders [15–18]. Previous studies have reported that self-management support significantly improved KTRs’ self-management skills, enhancing their medication adherence [15], and quality of life [19], underscoring the importance of offering self-management support in the care trajectory of KTRs.

However, Been-Dahmen [20] and Grijpma [21] found that KTRs’ needs for self-management support are not the same. They called for adequate tools to examine KTRs’ self-management support needs so that the medical staff can tailor self-management support interventions. Researchers have mentioned self-management support in a variety of ways, including, but not limited to, perceived support [20], received support [22] and provided support [23], among which the evaluation of support received by patients from their perspectives is expected to provide valuable information for future interventions. A scale to measure the amount of self-management support received and further identify patients’ unmet needs is necessary. However, such a scale for KTRs is lacking, leaving a field of research open for further exploration. To fill this gap, our study aimed to develop a Self-management Support Scale for Kidney Transplant Recipients (SMSSKTR) and test its psychometric properties.

## Methods

### Study design

**Fig. 1:**
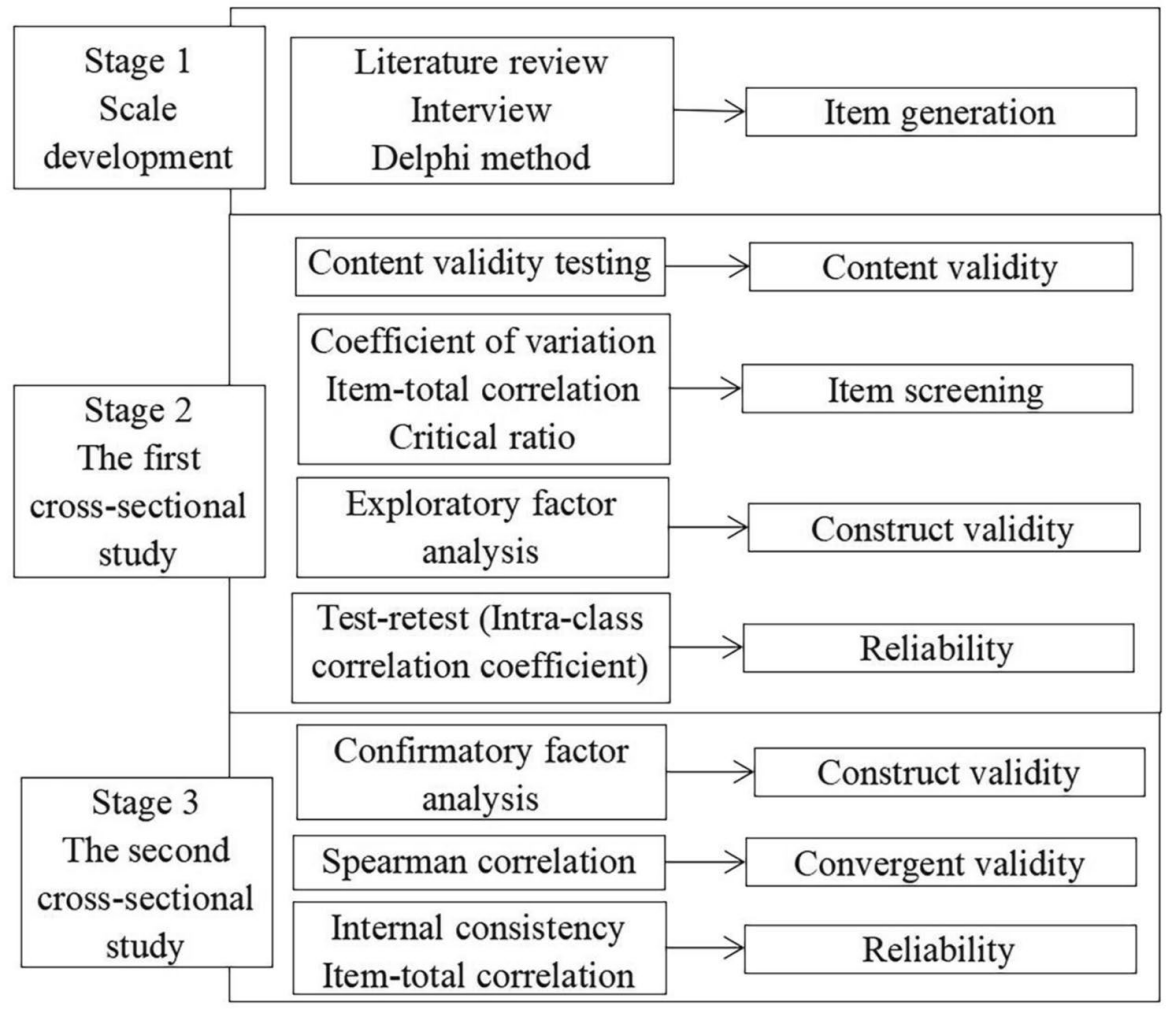
The flow chart for the multi-stage study design

This is an instrument development and validation study. We followed the recommendations for scale design and development by Rattray and Jones [24] and adapted a three-stage cross-sectional design. Stage 1 aimed at item generation based on relevant literature, scales, interview transcripts, and the Delphi method, generating the initial version of SMSSKTR. Next, two cross-sectional studies were conducted in Stage 2 and Stage 3 to test the validity and reliability of the scale (Fig. 1). We followed ‘Recommendations for reporting the results of studies of instrument and scale development and testing’ [25] for the layout of the paper, which combined the STARD and GRRAS checklist in the EQUATOR network.

### Stage 1: scale development

#### Item generation

Guided by the definition of self-management support from the ADA [13], we defined the categories and sources of support based on Wagner’s Chronic Care Model [26] and the thematic synthesis of self-management support for patients with chronic conditions by Dwarswaard et al. [14]. Next, we reviewed the literature and extracted relevant items from previous literature and relevant scales, such as the Patient Assessment of Chronic Illness Care (PACIC) [27] and Assessment of Chronic Illness Care (ACIC) [28]. We also interviewed the medical staff and KTRs to draw themes from interview transcripts to supplement the item pool. Once the initial instrument was constructed, the Delphi method was employed to examine whether the preliminary items accurately reflected the attributes of SMSSKTR. For the Delphi method, the inclusion criteria were experts who (1) had work experience with KTRs for no less than five years; (2) had expertise in kidney transplantation research or scale development; (3) held associated degree or above for nurses, master’s degree or above for physicians and pharmacists.

#### Sample/participants

Convenience sampling was used until data saturation was achieved. A total of 31 KTRs participated in the interview. Among them, 10 joined in the three focus group interviews and 21 joined in the one-on-one in-depth interviews. For interviewees, the inclusion criteria were KTRs (1) who were 18 years or older; (2) whose postoperative time was no less than three months, as KTRs would usually recover from the surgery and transit to the self-management phase after three months; (3) who could read and write Chinese; (4) who were oriented to person, place, time, and situation; and (5) who informed consented to participate in the study. Recipients of multiple kidney transplantation or combined multiple organ transplantations were excluded from the study, as their needs for self-management support are different from single-kidney transplantation recipients. We also selected medical staff (four doctors, seven nurses and one pharmacist) who provided follow-up services to KTRs, as they were aware of the self-support needs of those patients. They joined ten one-on-one in-depth interviews and one focus group interview.

#### Data collection

The data were collected between September 2018 and January 2019. One-on-one in-depth interviews or focus group interviews were conducted in a quiet room in China-Japan Friendship Hospital, Beijing. After participants signed the informed consent form, semi-structured interview guides were used to obtain rich data and achieve a better understanding of participants’ self-management support. The interview guide for patients included the following questions: (1) What difficulties do you face when managing your disease? (2) What support do you need when managing your disease? Where can you receive this support? The interview guide for the medical staff included the following questions: (1) What support do you think should be provided to KTRs? (2) What are the sources of support except for the medical staff? (3) What support do KTRs express during their clinical visits? All interviews were audio-recorded with permission and transcribed verbatim, and the main themes were extracted to form scale items. After the initial items were formed, a consultation form for the Delphi study was e-mailed to collect professional opinions on the dimensions and items of the scale. The importance of each item was rated on a 5-point Likert scale (1 = not important and 5 = very important). If an item was considered unsuitable, modification suggestions were expected.

### Stage 2: the first cross-sectional survey

#### Sample and setting

We invited six experts who had been engaged in kidney transplantation, self-management of chronic diseases, urology and other related research or clinical work for more than 10 years to evaluate the content validity of the scale. To verify construct validity using exploratory factor analysis (EFA), we selected 325 KTRs to fill in a questionnaire, as the sample size of 325 satisfied the minimum sample-to-item ratio of 5:1 for performing EFA [29]. The inclusion and exclusion criteria of the patients were consistent with those in Stage 1. Data were collected from the outpatient department of China-Japan Friendship Hospital between February 2019 and March 2019.

#### Instruments

##### Content validity evaluation form

This form was designed to collect experts’ opinions about each item in the initial SMSSKTR. The options were ‘not relevant = 1’, ‘somewhat relevant = 2’, ‘relevant = 3’, and ‘very relevant = 4’.

##### Demographic and clinical characteristics

We designed a general information questionnaire, including demographic information (age, gender, height, weight, education level, employment status, and marital status) and disease information (organ source). Height and weight were used to calculate the body mass index (BMI).

##### SMSSKTR-Initial version

It is a self-rated scale finalized in Stage 1, with a Likert-type response format ranging from 1(never) to 5(always). The total score ranges from 40 to 200. A higher score indicates more self-management support received by KTRs.

#### Data collection

The content validity evaluation forms, along with the initial SMSSKTR, were e-mailed to experts. They were given two weeks to rate the forms and return them. We decided to distribute the initial SMSSKTR to KTRs who met the inclusion criteria. Before the distribution, the investigators explained the purpose and procedure of this study to the participants and obtained informed consent from them. We informed them that the questionnaire was anonymous and that no personal information (e.g., names, addresses, and telephone) would be collected. The questionnaires were collected and checked immediately after they were completed. Incomplete information was requested to be supplemented. To test the stability of the scale, 30 KTRs were invited to fill in the initial SMSSKTR again two weeks after the completion of the first scale.

#### Data analysis

We calculated the content validity index (CVI) to assess the content validity of the scale. For each item, the number of experts scored on 3 or 4 point divided by the total number of experts is the CVI value of the item, while the number of items scored on 3 or 4 point by all experts divided by the total number of items is the CVI value of the scale [30]. To screen items of the initial scale, we calculated the coefficient of variation (items with coefficient ≥ 0.3 were retained) [31], item-total correlation coefficient (items with coefficient > 0.3 were retained) [32], and critical ratio. If the difference between the highest group (top 27%) and the lowest group (bottom 27%) of the total score did not reach the significance level (*p* < 0.05), the item would be deleted [33]. To test construct validity, we performed EFA using principal component analysis with varimax rotation. Factors were extracted based on eigenvalues value eguale to or higher than 1.00 and factor loadings eguale to or greater than 0.40 [34]. Before that, we performed a Kaiser-Meyer-Olkin (KMO) test and Bartlett’s test of sphericity to confirm the appropriateness of the EFA. The intra-class correlation coefficient (ICC) was calculated to indicate the test-retest reliability. ICC value < 0.5 indicates poor reliability, value between 0.5 and 0.75 indicates moderate reliability, value between 0.75 and 0.9 indicates good reliability, and values > 0.90 indicates excellent reliability [35]. IBM SPSS (version 20.0) was used for all the data analyses. All tests were two-sided, and a *p* value of less than 5% was considered statistically significant.

### Stage 3: the second cross-sectional survey

#### Sample and setting

Using convenience sampling, we selected KTRs from three kidney transplant follow-up centers. The inclusion and exclusion criteria for KTRs were the same as those in Stage 1. A total of 270 KTRs were recruited to complete the survey, which exceeded the suggested minimum sample size of 200 for confirmatory factor analysis (CFA) [36].

#### Instruments

##### Demographic and clinical characteristics

We collected the same demographic and clinical information as in Stage 2, which included demographic information (age, gender, height, weight, education level, employment status, and marital status) and disease information (organ source).

##### SMSSKTR-the second version

The second version of SMSSKTR was developed after the second EFA in Stage 2, which contained three dimensions and 22 items in total. The first dimension was instrumental support (nine items), the second dimension was psychosocial support (nine items), and the third dimension was relational support (four items). The scale is rated on a Likert-type response format ranging from 1(never) to 5(always). The total score ranged from 22 to 110. The higher the score, the more self-management support KTRs received.

##### Self-management scale for renal transplant recipients (SMSRTR)

Developed by Weng [37], this scale consists of three subscales and 27 items: problem-solving (10 items), partnership (4 items), and self-management behaviours (13 items). Responses ranged from 0 (never) to 10 (always). A higher score indicates better self-management of KTRs. The Cronbach’s alpha coefficients of the three subscales were 0.800, 0.700, and 0.810, respectively [37]. This scale was used for convergent validity analysis. Based on Sim’s study [38], we hypothesized that self-management support would be positively correlated with patients’ self-management.

#### Data collection

The data were collected between October 2020 and February 2021. All eligible KTRs were invited to fill in a questionnaire. After signing the informed consent form, participants completed the questionnaire. They were informed that the questionnaire was anonymous and that no personal information would be collected. The questionnaires were double checked when they were completed to minimize missing data.

#### Data analysis

We performed CFA to test the scale’s construct validity by using Mplus (Version 7.11). The model fit indices and cut-off points were selected based on Kline [39]: standardized chi-square statistics (χ^2^ / df; ＜5.0), comparative fit index (CFI; ≥0.9), Tucker-Lewis index (TLI; ≥0.9), root mean square error of approximation (RMSEA; ≤0.08), and standardized root mean square residual (SRMSR; ≤0.08). Convergent validity was examined using Spearman’s correlation coefficient between SMSSKTR and SMSRTR scores. Cronbach’s alpha coefficient and corrected item-total correlation coefficient were used to test the reliability of SMSSKTR. SPSS (version 20.0) was used for correlation and reliability analysis. All tests were two-sided, and a *p* value of less than 5% was considered statistically significant.

### Ethical consideration

In Stage 1, the interview process guaranteed the ethical criteria of data confidentiality, anonymity and voluntariness of the interviewees. In Stages 2 and 3, the informed consent form was signed before the participant filled in the questionnaire anonymously. All questionnaires and the informed consent forms were locked in a file cabinet. The transcripts and data files for analysis were stored safely in a password-protected computer.

## Results

### Results in stage 1

#### Participants’ characteristics and descriptive statistics

In our study, the average age of KTRs is 44 ± 13.25 years old. More than half of the patients were males (N = 20, 64.52%), and the average postoperative time was 4.79 ± 5.85 years. The average age for medical staff was 37.42 ± 8.34 years old, most of them were females (N = 9, 75%). The average working time in the kidney transplantation ward was 13.58 ± 7.74 years.

A total of 29 experts participated in the Delphi study. The average age was 42.24 ± 7.23 years. The working time in the kidney transplantation-related field was between five and thirty-four years. Among them, 18(62.1%) were nurses. 51.8% of the experts held a master’s degree or above.

#### Item extraction

We extracted 20 items related to self-management support for renal transplantation from the relevant literature, and extracted 27 items from the relevant scales. In addition, 39 items were extracted from interview transcripts of the patients and medical staff. We combined similar items and deleted overlapping items. Items that did not conform to the research topic were also removed, resulting in 36 items. After the two-round Delphi study, four items were deleted, eight items were added, 15 items were rephrased. For example, for the item “To what extent has your community organized health knowledge lectures or physical examinations for you after kidney transplantation?”, experts believed that it was not practical for the community to provide such services. Therefore, this item was deleted. When reviewing items in the relational support, experts believed that it should also include the support from peers. Therefore, items relevant to peer support was added (i.e., To what extent have your peers maintained a partnership with you and shared each other’s experience in the management of health condition after kidney transplantation). To avoid ambiguity, we also rephrased some items based on the feedback from experts. For example, for the item “To what extent has the medical staff given you daily guidance after kidney transplantation”, the concept of “daily guidance” was too abstract to be understood. Therefore, we added examples right after the term “daily guidance”. Finally, 40 items were achieved (Supplementary file).

### Results in stage 2

#### Participants’ characteristics and descriptive statistics

In this stage, 325 questionnaires were distributed, of which 12 were invalid due to missing data. Therefore, 313 questionnaires were valid and used for the analysis. The sample had an average age of 43.80 ± 11.20 years old, and an average BMI of 23.05 ± 3.54. More than half of the participants were males (N = 204, 65.18%). Most of the organs were from deceased donors (N = 256, 81.79%). Please refer to Table 1 for more details.

**Table 1:** Participants’ characteristics in Stage 2 & 3

	Stage 2(N = 313)				Stage 3(N = 265)		
	Category	n (%)	Mean	SD	n (%)	Mean	SD
Age			43.80	11.20		46.00	10.80
BMI			23.05	3.54		22.92	3.15
Gender	Female	109(34.82)			73(27.55)		
	Male	204(65.18)			192(72.45)		
Educational level	Junior high school and below	70 (22.36)			53(20.00)		
	Senior high school or diploma	90(28.76)			71(26.79)		
	College and above	153(48.88)			141(53.21)		
Employment status	Employed	153(48.88)			142(53.58)		
	Unemployed	160(51.12)			123(46.42)		
Marital status	Single/divorce	63(20.13)			47(17.74)		
	Married	250(79.87)			218(82.26)		
Organ source	Deceased donor	256(81.79)			241(90.94)		
	Living donor	57(18.21)			24(9.06)		

#### Content validity

The six experts rated to what extent each item in the initial SMSSKTR adequately reflected the theoretical definition of self-management support using the content validity evaluation form. We obtained CVI of 0.97 for the entire scale, with CVI values for each item ranging from 0.667 to 1.000.

#### Item screening

After we collected the responses of the participants regarding the initial SMSSKTR, we performed an analysis of the dispersion of items, correlation analysis, and discrimination analysis. Based on the criteria we set for item screening previously, we removed items 2, 11, 12, 13, 14, 15, 16, 17, 18, 19, 20, 23, 31, and 32, retained 26 items.

#### Construct validity by EFA

**Fig. 2:**
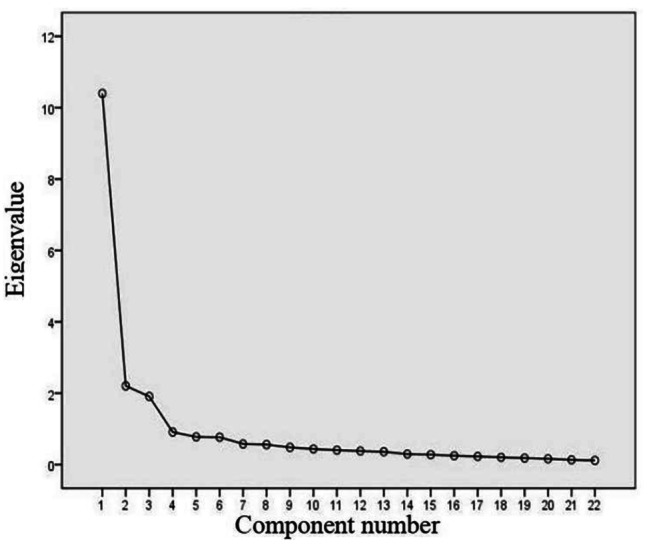
Scree plot for SMSSKTR after the second EFA

We used data from the 26-item SMSSKTR to perform the EFA. Bartlett’s test of sphericity (χ^2^ = 6549.871, *p* < 0.001) and the Kaiser-Meyer-Olkin value (0.929) attested to the adequacy of the correlation matrix for exploratory factor analysis. The scree plot analysis suggested a four-factor solution. We removed items 24, 38, 39, and 40 because their factor loadings were less than 0.40 (items 24 and 38) or because they had cross loadings (items 39 and 40). We performed the EFA again for the remaining 22 items. The KMO value was 0.929 and Bartlett’s test of sphericity was significant (χ^2^ = 5205.176, *p* < 0.001). The final EFA accounted for 66% of the total variance. All the 22 items were retained. The scree plot analysis suggested a three-factor solution (Fig. 2). We labelled the first factor as “instrumental Support” (nine items), which explained 25.9% of the total variance. We labelled the second factor as “psychosocial Support” (nine items), which explained 24.5% of the total variance. We labelled the third factor as “relational support” (four items), which explained 15.6% of the total variance. Table 2 shows the second EFA results of SMSSKTR.

**Table 2:** Three factors extracted from exploratory factor analysis using matrix rotation^a^

Item (To what extent have/has… after kidney transplantation?)	Factor 1	Factor 2	Factor 3
**Instrumental Support**			
1. the medical staff given you daily guidance (such as diet, exercise, disinfection and sanitation, etc.)	0.550		
2. the medical staff chosen the appropriate strengths of immunosuppressive medications based on your dose	0.701		
3. the medical staff given you medication guidance (such as modes of administration, actions taken for wrong taking, missing taking, and forgetting taking)	0.758		
4. the medical staff emphasized the requirements, do’s and don’ts for blood drug concentration examination to you	0.715		
5. the medical staff told you about the potential side effects of medications and alternative solutions	0.800		
6. the medical staff told you about the potential infections and how to prevent them	0.793		
7. the medical staff told you about potential adverse reactions (such as rejection) and alternative solutions	0.760		
8. the medical staff given you advice according to your physical conditions when you did not feel well	0.741		
9. the medical staff inquired about the cause and offer solutions when you did not follow instructions for treatments or medications	0.671		
**Psychosocial Support**			
10. your family, friends, and colleagues chosen foods that were good for your health		0.670	
11. your family, friends, and colleagues supervised you to take medications		0.561	
12. the medical staff provided psychological counselling when your kidney function did not recover or when there were complications		0.561	
13. your family, friends, and colleagues had a positive attitude towards the prognosis and management of your disease		0.730	
14. your family, friends, and colleagues offered emotional support when you were feeling down		0.807	
15. your family, friends, and colleagues encouraged you to stay positive		0.790	
16. communicating with peers built your confidence		0.680	
17. humorous communication helped you deal with your health condition more actively		0.800	
18.your family, friends, and colleagues shared with you your joys and sorrows		0.743	
**Relational Support**			
19. the medical staff asked you about your thoughts when making a treatment plan for you			0.875
20. the medical staff asked you about your health habits when making a rehabilitation plan for you			0.864
21. the medical staff or other caregivers listened carefully to your thoughts about your health condition			0.884
22. your family, friends, and colleagues showed understanding and support for the management of your health condition			0.738

a. Extraction method: principal component analysis

Rotation method: orthogonal rotation method with Kaiser standardization

The rotation converges after 5 iterations

#### Test-retest reliability of SMSSKTR

The intra-class correlation coefficient (ICC) for the entire scale was 0.915. The ICC for the three subscales were 0.771, 0.896, and 0.832, respectively. The results are presented in Table 3.

**Table 3:** Reliability demonstrated by intra-class correlation coefficient and 95% confidence intervals for SMSSKTR

Factors	ICC	95%CI	*P*-value
Instrumental Support	0.771	0.631–0.874	＜0.001
Psychosocial Support	0.896	0.833–0.943	＜0.001
Relational Support	0.832	0.722–0.909	＜0.001

### Results in stage 3

#### Participants’ characteristics and descriptive statistics

Of the 270 returned questionnaires, 265 were valid for analysis. The sample had an average age of 46.00 ± 10.80 years old, and an average BMI of 22.92 ± 3.15. More than half of the participants were males (N = 192, 72.45%). Most of the organs were from deceased donors (N = 241, 90.94%). Please refer to Table 1 for more details.

#### Construct validity by CFA

**Fig. 3:**
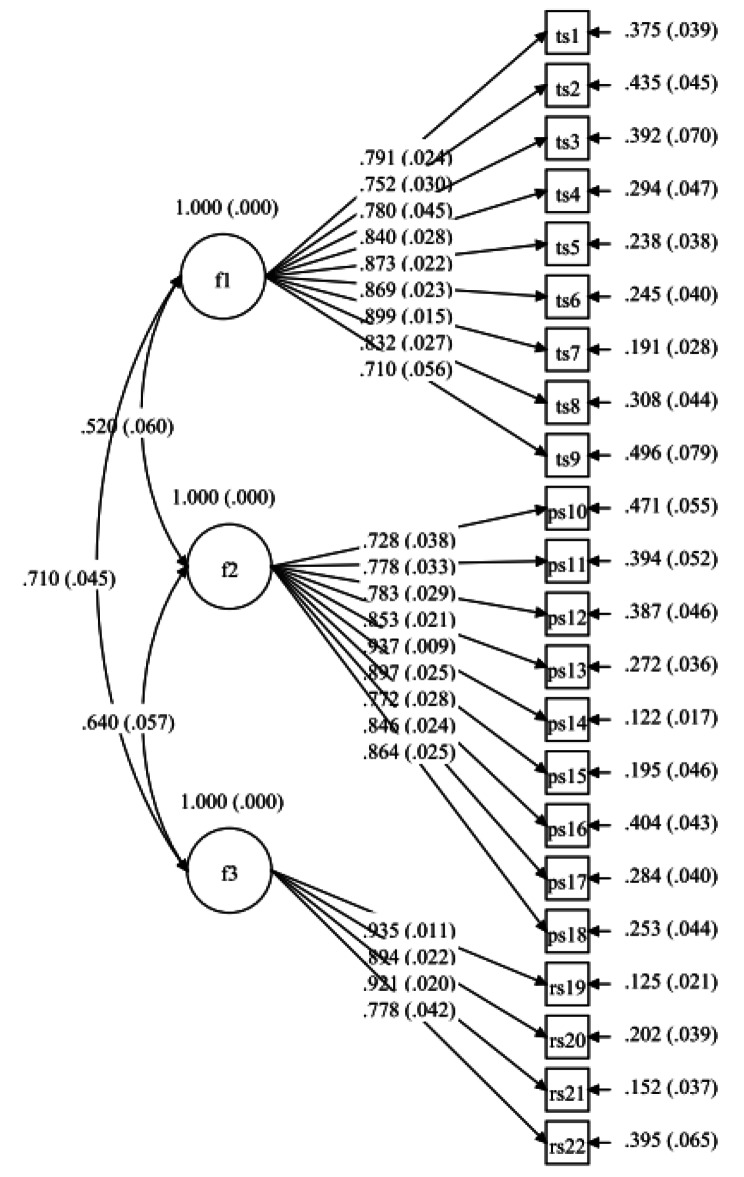
Standardized pathway coefficient plot after CFA for the final model^a^ a: f1 = Instrumental Support; f2 = Psychosocial Support; f3 = Relational Support

We used CFA to test the model with data from the 22-item SMSSKTR validated in Stage 2. The results showed that the CFA model achieved a good fit, with χ^2^ = 480.330, df = 206, χ^2^/df = 2.332, *p* < 0.001; CFI = 0.901; TLI = 0.889; RMSEA = 0.071, with a 90% confidence interval (0.063–0.079); and SRMR = 0.065 (Fig. 3).

#### Convergent validity

We found that the total score of SMSSKTR was positively associated with that of SMSRTR (*r* = 0.532, *p* < 0.01). Further details are provided in Table 4.

**Table 4:** Correlation coefficients between the scores of SMSSKTR and SMSRTR and their subscales

		b			
		Problem- solving	Partnership	Self-management behaviors	The entire scale
a	Instrumental Support	0.382*	0.410*	0.384*	0.442*
	Psychosocial Support	0.382*	0.369*	0.453*	0.469*
	Relational Support	0.402*	0.389*	0.419*	0.464*
	The entire scale	0.448*	0.451*	0.489*	0.532*

a: Self-Management Support Scale for Kidney Transplant Recipients (SMSSKTR).

b: Self-Management Scale for Renal Transplant Recipients (SMSRTR).

* *p* < 0.01.

#### Reliability

The internal consistency of SMSSKTR was tested by calculating Cronbach’s alpha coefficient, which was 0.959 for the entire scale and 0.956 to 0.958 for the three subscales. The corrected item-total correlation coefficient ranged from 0.62 to 0.82.

## Discussion

Assessing the self-management support received by KTRs helps medical staff to identify self-management support KTRs lack and tailor interventions accordingly. The purpose of this study was to develop and psychometrically evaluate an instrument to assess received self-management support for KTRs, namely, SMSSKTR. We followed the recommendations of Rattray and Jones [24] to ensure that the scale was scientifically developed. The final product is a 5-point Likert scale that includes three dimensions and 22 items. The total score ranges from 22 to 110, with higher scores indicating more self-management support received by KTRs. SMSSKTR is one of the first instruments with adequate psychometric properties to evaluate self-management support received by KTRs.

The construct validity of SMSSKTR was evaluated by EFA, followed by CFA. The results of the EFA revealed a three-factor structure explaining 66% of the total variance, which was within the recommended range for multidimensional scales [40]. This indicates that SMSSKTR provides adequate coverage for evaluating the self-management support received by KTRs. This structure was further confirmed by CFA, which met our expectation about the structure of SMSSKTR. In Stage 1, we proposed the definition of self-management support for KTRs and its dimensions according to ADA [13], the Chronic Care Model [26], and the thematic synthesis of self-management support for patients with chronic conditions [14]. Using the definition and dimensions as a guide, we formed the initial items by extracting them from the literature, relevant scales and themes of interview transcripts. Next, we invited experts for the suitability of the items and performed a statistical analysis to screen items of the initial scale. All of these steps ensured the theoretical soundness of SMSSKTR. The three-factor structure indicated that the self-management support of KTRs was a multifaceted and multidimensional concept, which was in line with the current trends of integration of the full care team for chronic diseases [41]. Our results showed that the score of SMSSKTR, SMSRTR and their subscales were positively correlated, which was consistent with the finding of Sim et al. [38] and our hypothesis that healthcare professionals’ support was positively correlated with patients’ self-management, providing evidence for the convergent validity.

Factor 1 is labelled “instrumental support”, referring to disease-related medical management KTRs received from the medical staff. This dimension includes nine items accounting for 25.9% of the total variance, covering topics such as received support for daily life care and self-management of post-transplantation complications, treatment plans, and side effects of medication. As mentioned earlier, KTRs must deal with rejection and complications induced by transplantation. Therefore, medication management is a key element. However, Ranahan et al. [42] found that very few KTRs felt confident when explaining how their medications worked, expressing that this information ‘was above their head’, and many felt unprepared for the pill burden, side effects, doses, and medication management encountered after transplantation. These findings highlight the importance of receiving medical management support. Our scale offers a way to check the extent to which KTRs have already received this kind of support and therefore, identify their unmet needs.

Factor 2 is labelled “psychosocial support”, referring to the emotional and psychological resources needed to manage the disease. This dimension includes nine items and accounts for 24.5% of the total variance, asking about the extent to which KTRs have received this kind of support from a variety of sources, not only from professionals but also from people around them, such as peers, family, friends, and colleagues. As suggested by Chisholm-Burns et al. [43], involving family members and/or friends as a support system would facilitate self-management adherence. Therefore, the support sources should vary. As pointed out by Been-Dahmen et al. [20], KTRs’ emotional and social support needs were usually overlooked. Rating the nine items in the psychosocial support dimension would not only check the amount of psychosocial support received but also raise the awareness of the medical staff regarding providing this kind of support.

Factor 3 is labelled “relational support”, referring to beneficial interaction with others. Although this dimension has only four items and accounts for 15.6% of the total variance, it is the centre of patients’ support needs and provides motivation for other types of support [14]. On one hand, the medical staff show respects towards KTRs when making plans; On the other hand, medical staff, family, friends, and colleagues show understanding and support towards KTRs. It is necessary to consider patients’ thoughts when developing a treatment plan, which is beneficial to improve KTRs’ adherence [43]. One-way communication hinders effective partnerships necessary for disease management [44]. The four items in the relational support dimension help identify the extent to which KTRs have received this kind of beneficial interaction.

As for the reliability, Cronbach’s alpha coefficient for the entire scale was 0.959 and ranged from 0.956 to 0.958 for its three subscales, showing excellent internal consistency of SMSSKTR. The corrected item-total correlation coefficient ranged from 0.62 to 0.82, indicating acceptable associations. The ICC value reached 0.915 when assessing the test-retest reliability, indicating excellent reliability and time stability [45].

## Strength and limitation

To the best of our knowledge, SMSSKTR is one of the first instruments to assess the self-management support received by KTRs. We followed the recommendations for scale design and development proposed by Rattray and Jones [24] to ensure scientific development and validation procedures. However, this study has several major limitations. First, due to the difficulties of following up participants during the COVID-19 pandemic, data for calculating the test-retest reliability were missing in Stage 3. We can only rely on the data obtained in Stage 2 instead of Stage 3 to calculate the test-retest reliability. Fortunately, we had 30 KTRs to fill in SMSSKTR twice in Stage 2, and the 22 items supposed to be used to calculate the test-retest reliability remained the same in the two stages, which made the calculation possible and scientific. Second, the participants responded to questionnaires during their outpatient visits. This might have affected their answers to the items because of their busy schedules. Thirdly, although the sample size in our study was sufficient to perform EFA and CFA, it was not large enough to carry out the statistical analysis to set the cutoff point for SMSSKTR. Further research is required in this regard. Finally, we developed the scale based on Wagner’s Chronic Care Model. Therefore, items regarding digitalization/eHealth were removed when the scale was finalized. It would be of great importance to modify our scale based on the eHealth Enhanced Chronic Care Model [46] in future study to reflect this trend.

## Conclusion

Using the three-stage design, our study shows that SMSSKTR has good reliability and validity, indicating that it can be used as an evaluation tool to measure the amount of self-management support received and clarify what self-management support KTRs lack, thus providing guidance for medical staff, families, friends, and colleagues to support KTRs in a timely and targeted manner, and ultimately improving their self-management and health outcomes. Given the general nature of the items in SMSSKTR, it will be of great significance to validate this scale to other transplant patient groups in the future.

## Electronic supplementary material

Below is the link to the electronic supplementary material.

Supplementary Material 1

Supplementary Material 2

## Data Availability

The datasets used during the current study are available from the corresponding author upon request.

## Abbreviations

ADA: American Diabetes Association
KTRs: kidney transplant recipients
SMSSKTR: Self-management Support Scale for Kidney Transplant Recipients
PACIC: Patient Assessment of Chronic Illness Care
ACIC: Assessment of Chronic Illness Care
EFA: exploratory factor analysis
BMI: body mass index
CVI: content validity index
KMO: Kaiser-Meyer-Olkin
ICC: the intra-class correlation coefficient
CFA: confirmatory factor analysis
SMSRTR: Self-Management Scale for Renal Transplant Recipients
CFI: comparative fit index
TLI: Tucker-Lewis index
RMSEA: root mean square error of approximation
SRMSR: standardized root mean square residual
IRB: Institutional Review Board
