# Supplementary material for: Development and validation of the Chinese version of the self-management support scale for kidney transplant recipients
Source: BMC Nurs. 2023 Apr 18;22:128. doi: 10.1186/s12912-023-01269-x (PMC10111319; doi:10.1186/s12912-023-01269-x)
Supplement: Supplementary file 1 — Supplementary Material 1 [file 12912_2023_1269_MOESM1_ESM.docx]

**Supplementary file**

**Development and validation of the Chinese version of the Self-Management Support Scale for Kidney Transplant Recipients**

Table The initial version of the Self-Management Support Scale for Kidney Transplant Recipients^a^

| Dimension | Item (To what extent have/has... after kidney transplantation?) |
| --- | --- |
| Instrumental support | 1.the medical staff given you daily guidance (such as diet, exercise, disinfection, and sanitation) |
|  | 2.the medical staff taught you family members common knowledge of your disease (such as diet, exercise, disinfection, and sanitation) |
|  | 3.the medical staff chosen the appropriate strengths of immunosuppressive medications based on your dose |
|  | 4.the medical staff given you medication guidance (such as modes of administration, actions taken for wrong taking, missing taking, and forgetting taking) |
|  | 5.the medical staff emphasized the requirements, do’s and don’ts for blood drug concentration examination to you |
|  | 6.the medical staff told you about the potential side effects of medications and alternative solutions |
|  | 7.the medical staff told you about the potential infections and how to prevent them |
|  | 8.the medical staff told you about the potential adverse reactions (such as rejection) and alternative solutions |
|  | 9.the medical staff given you advice according to your physical condition when you did not feel well |
|  | 10.the medical staff inquired about the cause and offered solutions when you did not follow the instructions for treatments or medications |
|  | 11.the medical staff followed up regularly via telephone or WeChat App |
|  | 12.the hospital set up an online or outpatient electronic reservation system |
|  | 13.the hospital provided an online Q&A platform (such as WeChat group, official account, and forum) |
|  | 14.the hospital held regular learning sessions (such as kidney transplantation recipients club and lectures) |
|  | 15.the hospital provided you with professional renal transplantation follow-up services |
|  | 16.medical insurance covered your expenses of kidney transplantation |
|  | 17.the pharmacy provided you with the medicine you needed |
|  | 18.your community had suitable fitness facilities or places for you |
|  | 19.professional websites or medical books provided you with information related to health management |
|  | 20.your family, friends, and colleagues urged you or accompanied you to exercise |
|  | 21.your family, friends, and colleagues chosen foods that were good for your health |
|  | 22.your family, friends, and colleagues supervised you to take medications |
|  | 23.your family, friends, and colleagues helped you when you needed help or when you did not feel well |
|  | Item (To what extent have/has... after kidney transplantation?) |
| Psychosocial Support | 24.the medical staff acknowledged and encouraged your efforts in health management |
|  | 25.the medical staff provided psychological counselling when your kidney function did not recover or when there were complications |
|  | 26.your family, friends, and colleagues had a positive attitude towards the prognosis and management of your disease |
|  | 27.your family, friends, and colleagues offered emotional support when you were feeling down |
|  | 28.your family, friends, and colleagues encouraged you to stay positive |
|  | 29.communicating with peers built your confidence |
|  | 30.humorous communication helped you deal with your health condition more actively |
|  | 31.your community given you material and emotional support and help |
|  | 32.the activities held in your community made you happy |
|  | 33.your family, friends, and colleagues shared with you your joys and sorrows |
|  | Item (To what extent have/has... after kidney transplantation?) |
| Relational Support | 34.the medical staff asked you about your thoughts when making a treatment plan for you |
|  | 35.the medical staff asked you about your health habits when making a rehabilitation plan for you |
|  | 36.the medical staff or other caregivers listened carefully to your thoughts about your health condition |
|  | 37.your family, friends, and colleagues showed understanding and support for the management of your health condition |
|  | 38.your family, friends, and colleagues listened carefully to your views towards the management of your health condition |
|  | 39.your peers understood your confusion about the management of kidney transplantation and given you advice |
|  | 40.your peers maintained a partnership with you and shared each other's experience in the management of health condition |

 a: The options for each item are Never, Seldom, Sometimes, Often, and Always
